# Effectiveness of trauma centers verification: Protocol for a systematic review

**DOI:** 10.1186/s13643-019-1239-6

**Published:** 2019-11-28

**Authors:** Brice Batomen, Lynne Moore, Mabel Carabali, Pier-Alexandre Tardif, Howard Champion, Arijit Nandi

**Affiliations:** 1 Department of Epidemiology, Biostatistics, and Occupational Health | McGill University, Charles Meredith House, 1130 Pine Avenue West, Room B9, Montreal, Quebec H3A 1A3 Canada; 2 Department of Social and Preventative Medicine, Université Laval, Quebec City, Quebec Canada; 3 Population Health and Optimal Health Practices Research Unit, Trauma–Emergency–Critical Care Medicine, Centre de Recherche du CHU de Québec–Université Laval (Hôpital de l’Enfant-Jésus), Université Laval, Quebec City, Quebec Canada; 4 Department of Surgery, Uniformed Services University of the Health Sciences, Bethesda, MD USA; 5 Institute for Health and Social Policy, Department of Epidemiology, Biostatistics, and Occupational Health | McGill University, Montreal, Quebec Canada

**Keywords:** Emergency health service, Trauma centers, Healthcare delivery, Hospital policy, Guideline adherence

## Abstract

**Background:**

The implementation of trauma systems in many high-income countries over the last 50 years has led to important reductions in injury mortality and disability in many healthcare jurisdictions. Injury organizations including the American College of Surgeons and the Trauma Association of Canada as well as the World Health Organization provide consensus-based recommendations on resources and processes for optimal injury care. Many hospitals treating trauma patients seek verification to demonstrate that they meet these recommendations. This process may be labeled differently across jurisdictions. In Canada for example, it is called accreditation, but it has the same objective and very similar modalities. The objective of the study described in this protocol is to systematically review evidence on the effectiveness of trauma center verification for improving clinical processes and patient outcomes in injury care.

**Methods:**

We will perform a systematic review of studies evaluating the association between trauma center verification and hospital mortality (primary outcome), as well as morbidity, resource utilization, and processes of care (secondary outcomes). We will search CINAHL, EMBASE, HealthStar, MEDLINE, and ProQuest databases, as well as key injury organization websites for gray literature. We will assess the methodological quality of studies using the Risk Of Bias In Non-randomized Studies – of Interventions (ROBINS-I) assessment tool. We are planning to conduct a meta-analysis if feasible based on the number of included studies and their heterogeneity. We will evaluate the quality of cumulative evidence and strength of recommendations using the Grading of Recommendations Assessment, Development and Evaluation (GRADE) working group methodology.

**Discussion:**

This review will provide a synthesis of the body of evidence on trauma center verification effectiveness. Results could reinforce current verification modalities and may suggest ways to optimize them. Results will be published in a peer-reviewed journal and presented at an international clinical conference.

**Systematic review registration:**

PROSPERO CRD42018107083**.**

## Background

Injuries represent an estimated 15% of the global burden of disease [1]. They are the leading cause of death under 40 years of age in North America [2–4]. The implementation of trauma systems in many high-income countries over the last 50 years has led to important reductions in injury mortality, disability, and overall costs in many healthcare jurisdictions [5–7]. Many injury organizations, most notably the American College of Surgeons (ACS) [8], provide consensus-based recommendations on the structure of trauma systems, and there is a growing trend towards verification of hospitals within trauma systems to determine if they meet criteria for optimal care. Trauma center accreditation is a similar process [9, 10]. We will hereafter use the term “verification” to refer both to accreditation and verification.

Generally, hospitals are designated as trauma centers before applying for verification. Trauma center designation is conducted by a regional or provincial health authority at a local or state level. Designation criteria and procedures may vary from state to state and are typically outlined through the legislative or regulatory authority [11]. Designated hospitals may then seek verification with organizations such as the ACS, which assess adherence to recommended care related to resources, commitment, readiness, policies, patient care, and performance improvement [10].

ACS verification is generally accomplished in two steps: (1) hospitals submit a prereview questionnaire, which allows site reviewers to have a preliminary understanding of the trauma care capabilities, and (2) a peer review team nominated by the College conducts an on-site review of the hospital [10, 12, 13]. During the process, a center is evaluated according to its designation level. Perceived advantages of verification include strengthening partnerships with stakeholders, engagement and commitment, team work, and the identification of improvement opportunities and priorities [8, 14]. However, verification is an expensive and resource-consuming process [15, 16]. A recent study in the Georgia trauma system estimated that the average costs of American College of Surgeon verification readiness (including administrative resources, clinical medical staff, in-house operating room, and education/outreach) for level I and level II trauma centers were $6.8 and $2.3 million respectively [17]. It is essential to know if verification is a good investment of money and resources in terms of improving patient outcomes [16, 18]. We lack a systematic evidence synthesis on the effectiveness of trauma center verification.

This review aims to synthesize evidence on the effectiveness of trauma center verification for improving hospital mortality, morbidity, resource utilization, and processes of care.

## Methods

The protocol is developed and presented using the structure suggested by Preferred Reporting Items for Systematic review and Meta-Analysis protocols (PRISMA-P) [19] (Additional file 1). It has been registered in the International Prospective Register of Systematic Reviews (PROSPERO) database, **CRD42018107083** **[**20**].** Any important protocol amendments will be reported and justified in the subsequent systematic review manuscript.

### Participants and study designs

Our study population consists of injured patients treated at trauma centers. We will include randomized and non-randomized controlled trials, quasi-experimental studies, controlled before-after studies, cross-sectional studies, and prospective or retrospective observational studies. Case reports, case series, and narrative studies which do not provide an estimate of the association between verification and the investigated outcomes will be excluded. No geographical area, language, or date of publication restrictions will be applied.

### Interventions and comparators

The intervention under evaluation is trauma center verification (Table 1). Comparison groups will be non-verified centers or the same center before it was verified.

**Table 1:** PICO statement

Population	Trauma patients treated in trauma centers
Intervention	Successful verification
Comparison	Non-verified trauma centers or the same center before verification
Outcomes	Mortality, morbidity, resource utilization, processes of care

### Outcome measures

The primary outcome is hospital mortality. Secondary endpoints include morbidity (e.g., complication), resource utilization (e.g., length of stay, costs), and adherence to evidence-based processes of care (e.g., venous thromboembolism prophylaxis).

### Search strategy and data sources

The search strategy will be developed by information specialists using appropriate Boolean operators to combine keywords and controlled vocabulary. Keywords will be identified by a group of experts and will be based on a combination of the terms “accreditation/verification” and “injury/trauma.” Cumulative Index to Nursing and Allied Health Literature (CINAHL), Excerpta Medica dataBASE (EMBASE), HealthStar, MEDLINE, and ProQuest Dissertation & Theses databases will be searched. Moreover, the websites of key injury organizations will be screened^1^. A preliminary version of the search strategy is presented in Additional file 2.

### Data management

References will be managed using EndNote software (version X9, New York City: Thomson Reuters, 2018). Duplicates will be identified and eliminated using a peer-reviewed published approach which consists of electronic and manual screening [21]. Multiple publications based on the same data will be identified by crosschecking authors, dates, and settings. Only one publication will be kept for analyses using criteria based on study dates (most recent), sample size (largest), and risk of bias (lowest risk).

### Selection process

To ensure consistency in study selection, reviewers will evaluate two to three sets of 100 randomly identified records. Once satisfactory inter-rater agreement has been reached (Kappa score > 0.7) between the two reviewers (BB, MC), they will independently review titles and abstracts of all identified records by applying the inclusion and exclusion criteria to select potentially eligible studies for full-text review. Full texts of the latter will then be retrieved and examined to determine eligibility. Discrepancies will be resolved by consultation with a third reviewer (LM). Study selection process as well as reasons for exclusions of potentially eligible studies will be described using a PRISMA flow chart.

### Data collection

Using a piloted standardized extraction form, two reviewers (BB, MC) will independently extract the following information: study reference, setting, design, years of the study, sample size (hospitals and patients), characteristics of the study sample (age, injury type), organizations responsible for verification, outcomes (including details of units of measurement), measures of associations for each outcome as reported, and covariates adjusted for. If information is available solely in figures, a computer-assisted program will be used to extract graphical data [22, 23]. Corresponding authors of included studies will be contacted by email (up to three attempts) if the aforementioned data are not reported.

### Risk of bias

Quality appraisal will be conducted by two independent trained reviewers (BB, MC) with content and methodological expertise in epidemiology, statistics, and trauma. Discrepancies will be resolved by discussion with a third reviewer (LM). The Risk Of Bias In Non-randomized Studies – of Interventions (ROBINS-I) assessment tool will be used to assess the methodological quality of studies and risk of bias [24]. If any RCT is included, the revised Cochrane risk of bias tool for randomized trials (RoB 2) will be used [25]. Both tools will be piloted on a random sample of 5% of the included studies to ensure consistency among reviewers.

### Data synthesis

Characteristics of primary studies will be presented using a table and described narratively. If more than two studies have evaluated the same category of outcome (hospital mortality for example), a meta-analysis will be performed [26]. Pooled effect estimates and 95% confidence intervals will be calculated using random effects models weighted by the inverse of the variance of estimates. Publication bias will be explored using funnel plots [27]. The quality of evidence and strength of recommendations will be evaluated using the Grading of Recommendations Assessment, Development and Evaluation (GRADE) working group methodology [28]. We will present results using a narrative synthesis if heterogeneity across studies in terms of populations, design, or methods is too great.

### Subgroup and sensitivity analyses

If data are available, analyses will be conducted by trauma center designation level. Previous studies have indicated that patients taken to level I centers have improved survival and better functional outcomes compared with those taken to level II centers [29, 30]. We will also present the results stratified by study years (in decades) to account for the evolution of the verification process since its introduction, by geographical regions (North America, Europe, Asia, Africa, and Australia), and by risk of bias (low, medium, and high), in line with the PRISMA statement [31].

### Timeframe for conducting the review

We are planning to complete the review and submit it for publication within 1 year of protocol registration on PROSPERO. An update will be undertaken if more than 6 months separate the date of the last search and the date of submission for publication.

## Discussion

This review is being undertaken as part of a research project aiming to advance knowledge on characteristics of the verification process that could optimize its effectiveness, including the optimal frequency of verification visits.

Verification of trauma centers is currently used in the USA and in many other high-income countries on the basis that it strengthens partnerships with stakeholders, team work, and identification of improvement opportunities and priorities [8, 14]. Therefore, synthesizing available evidence on its effectiveness represents an important step towards improving the management of trauma systems and consequently injury outcomes. The results of this review will fill an important knowledge gap and will provide information which may reinforce current verification modalities and may suggest ways to optimize them.

Our proposed systematic review is based on state-of-the-art methodological and reporting standards [19, 24, 28]. However, despite our intention to employ widely accepted statistical models for meta-analysis, elevated statistical heterogeneity in most of our planned analyses is expected. We anticipate this variability due to several factors, the most notable being that we expect that most studies will be based on observational data. Finally, verification is a complex health intervention and we expect substantial heterogeneity in intervention characteristics among eligible studies, due to the evolution of the verification process since its introduction and geographical variations.

We plan to disseminate our results via presentation at international clinical conferences and by publishing the results in a peer-reviewed journal.

## Supplementary information

**Additional file 1.** PRISMA-P (Preferred Reporting Items for Systematic review and Meta-Analysis Protocols) 2015 checklist: recommended items to address in a systematic review protocol.

**Additional file 2.** Preliminary search strategy.

## Data Availability

The datasets that will be generated and analyzed during the review will be available from the corresponding author on reasonable request.

## Abbreviations

ACS: American College of Surgeons
CINAHL: Cumulative Index to Nursing and Allied Health Literature
EMBASE: Excerpta Medica dataBASE
GRADE: Grading of Recommendations Assessment, Development and Evaluation
PRISMA-P: Preferred Reporting Items for Systematic review and Meta-Analysis protocols
PROSPERO: International Prospective Register of Systematic Reviews
ROBINS-I: The Risk Of Bias In Non-randomized Studies – of Interventions
WHO: World Health Organization

## Footnotes

1 The American College of Surgeons, Trauma Association of Canada, International Association for Trauma Surgery and Intensive Care, Australasian Trauma Society, Trauma Audit Research Network, American Association for the Surgery of Trauma, Eastern Association for the Surgery of Trauma, American Trauma Society, British Trauma Society, Orthopaedic Trauma Association, Western Trauma Association, Trauma.org, The Society of Trauma Nurses, International Trauma Anaesthesia and Critical Care Society, Brain Trauma Foundation
