# Supplementary material for: Effectiveness of trauma centers verification: Protocol for a systematic review
Source: Syst Rev. 2019 Nov 28;8:292. doi: 10.1186/s13643-019-1239-6 (PMC6882165; doi:10.1186/s13643-019-1239-6)
Supplement: Supplementary file 2 — Additional file 2. Preliminary search strategy. [file 13643_2019_1239_MOESM2_ESM.docx]

**Preliminary search strategy**

**EMBASE**

1. exp emergency health service/

2. (trauma adj (system* or care or network* or health care or model* or center* or centre* or service*)).tw,kf

3. (injury adj (system* or care or network* or health care or model* or center* or centre* or service*)).tw,kf.

4. 1 or 2 or 3

5. quality assuran*/ or quality improv*/ or benchmark*/ or clinical audit*.tw,kf. or medical audit*.tw,kf.

6. licensing/ or professional standard/ or certification/ or recertification/

7. "american college of surgeon*".tw,kf.

8. "trauma association of canada".tw,kf.

9. accreditation canada.tw,kf.

10. INESSS.tw,kf.

11. "Institut national d'excellence en santé et en services sociaux".tw,kf.

12. (designated or designation or designate).tw,kf.

13. (accredited or accreditation).tw,kf.

14. (verified or verification or reverification).tw,kf.

15. 5 or 6 or 7 or 8 or 9 or 10 or 11 or 12 or 13 or 14

16. 4 and 15

17. remove duplicates from 16

**MEDLINE**

| 1. exp Trauma Centers/ |
| --- |
| 2. (trauma adj (system* or network* or care or health care or model* or center* or centre* or service*)).tw,kf. |
| 3. (injury adj (system* or care or network* or health care or model* or center* or centre* or service*)).tw,kf. |
| 4. 1 or 2 or 3 |
| 5. exp Benchmarking/ |
| 6. clinical audit/ or exp medical audit/ |
| 7. quality assuran*/ or quality improv*/ or benchmark*/ or clinical audit*.tw,kf. or medical audit*.tw,kf. |
| 8. (accredited or accreditation).tw,kf. |
| 9. (verified or verification or reverification).tw,kf. |
| 10. "american college of surgeon*".tw,kf. |
| 11. "trauma association of canada".tw,kf. |
| 12. accreditation canada.tw,kf. |
| 13. INESSS.tw,kf. |
| 14. "Institut national d'excellence en santé et en services sociaux".tw,kf. |
| 15. (designated or designation or designate).tw,kf. |
| 16. 5 or 6 or 7 or 8 or 9 or 10 or 11 or 12 or 13 or 14 or 15 |
| 17. 4 and 16 |
| 18. remove duplicates from 17 |

**HEALTHSTAR**

| 1. Trauma Centers/ |
| --- |
| 2. (trauma adj (system* or care or network* or health care or model* or center* or centre* or service*)).tw,kf. |
| 3. (injury adj (system* or care or network* or health care or model* or center* or centre* or service*)).tw,kf. |
| 4. 1 or 2 or 3 |
| 5. Benchmarking/ |
| 6. clinical audit/ or exp medical audit/ |
| 7. quality assuran*/ or quality improv*/ or benchmark*/ or clinical audit*.tw,kf. or medical audit*.tw,kf. |
| 8. accreditation/ or certification/ or licensure/ |
| 9. (accredited or accreditation).tw,kf. |
| 10. (verified or verification or reverification).tw,kf. |
| 11. "american college of surgeon*".tw,kf. |
| 12. "trauma association of canada".tw,kf. |
| 13. accreditation canada.tw,kf. |
| 14. INESSS.tw,kf. |
| 15. "Institut national d'excellence en santé et en services sociaux".tw,kf. |
| 16. (designated or designation or designate).tw,kf. |
| 17. 5 or 6 or 7 or 8 or 9 or 10 or 11 or 12 or 13 or 14 or 15 or 16 |
| 18. 4 and 17 |
| 19. remove duplicates from 18 |

**CINAHL**

| S1 | TI ( trauma N1 (system* or network* or care or "health care" or model* or center* or centre* or service*) ) OR AB ( trauma N1 (system* or network* or care or "health care" or model* or center* or centre* or service*) ) |
| --- | --- |
| S2 | TI ( injury N1 (system* or network* or care or "health care" or model* or center* or centre* or service*) ) OR AB ( injury N1 (system* or network* or care or "health care" or model* or center* or centre* or service*) ) |
| S3 | S1 OR S2 |
| S4 | (MH "Accreditation+") OR (MH "American Accreditation Healthcare Commission") |
| S5 | ""''accredited'' OR "accreditation""" |
| S6 | (MH "Quality Assurance") OR "''clinical audit'' or ''quality assurance'' |
| S7 | (MH "Benchmarking") OR "benchmarking" OR (MH "Process Assessment (Health Care)+") |
| S8 | ""designation"" |
| S9 | ""American College of Surgeons"" |
| S10 | ""Trauma Association of Canada"" |
| S11 | ""Accreditation Canada"" |
| S12 | ""INESSS"" |
| S13 | ""Institut national d'excellence en santé et en services sociaux"" |
| S14 | S4 OR S5 OR S6 OR S7 OR S8 OR S9 OR S10 OR S11 OR S12 OR S13 |
| S15 | S3 AND S14 |

**ProQuest Dissertations & Theses Global**

((ti(accredited OR accreditation) OR ab(accredited OR accreditation)) OR (ti(verified OR verification OR reverification) OR ab(verified OR verification OR reverification)) OR (ti("clinical audit" OR benchmarking) OR ab("clinical audit" OR benchmarking)) OR (ti(designation OR designated) OR ab(designation OR designated)) OR (ti("American College of Surgeons") OR ab("American College of Surgeons")) OR (ti("Trauma Association of Canada") OR ab("Trauma Association of Canada")) OR (ti("Accreditation Canada") OR ab("Accreditation Canada")) OR (ti("Institut national d'excellence en santé et en services sociaux") OR ab("Institut national d'excellence en santé et en services sociaux")) OR (ti("INESSS") OR ab("INESSS"))) AND ((ti(trauma NEAR/1 (system* OR network* OR care OR "health care" OR model* OR center* OR centre* OR service*)) OR ab(trauma NEAR/1 (system* OR network* OR care OR "health care" OR model* OR center* OR centre* OR service*))) OR (ti(Injur* NEAR/1 (system* OR network* OR care OR "health care" OR model* OR center* OR centre* OR service*)) OR ab(Injur* NEAR/1 (system* OR network* OR care OR "health care" OR model* OR center* OR centre* OR service*))))
